# A chronological study on formation mechanism of nesquehonite from nanoparticles to grown crystals and its application in nanoparticle synthesis

**DOI:** 10.1038/s41598-025-04662-5

**Published:** 2025-07-01

**Authors:** Amin Yahyazadeh, Ali Faham Mofrad, Ehsan Yahyazadeh, Mohammad Outokesh, Seyed Mohammadreza Safavi, Sina Kazzazi, Neda Madani, Behzad Badr Hesari

**Affiliations:** 1 Department of Energy Engineering, Sharif University of Technology, Azadi Ave, P.O. Box: 11115-1639, Tehran, Iran; 2 School of Chemistry, College of Science, University of Tehran, Enghelab Ave, P.O. Box: 14155-6619, Tehran, Iran; 3 Kani Ara Sirjan Company, No 4, Eastern Atefi Ave., Africa Blv, Tehran, Iran

**Keywords:** Nesquehonite, Formation mechanism, Nucleation and growth, Induction period, Capping agent, Materials chemistry, Chemical engineering

## Abstract

Nesquehonite or hydrated magnesium carbonate is an ideal precursor for the production of magnesium compounds. One of the most important industrial routes for the synthesis of Nesquehonite is the reaction of MgSO_4_ (or MgCl_2_), existing in natural or desalination brines with Na_2_CO_3_. During this reaction, the viscosity (and other bulk properties) of the slurry dramatically increases after 20–40 min induction period. Such a surge of viscosity is important for the reactor engineers, as it may damage the driving motor. The current study was undertaken to 1: Elucidate the formation mechanism of the Nesquehonite crystals and its induction period, and 2: Propose a method for the production of nano MgCO_3_ by stopping the formation reaction in its early stage. By simultaneous monitoring of the microstructure and bulk properties using SEM, XRD, FTIR, Raman, TGA, and Rheometry, the following formation mechanism was suggested: The nano-sized nuclei of “MgCO_3_·3H_2_O” are formed, nearly instantaneously after contacting the reagents. Those nanoparticles need an induction period to form the sheet-like intermediate. Large crystals are then formed quickly through stacking of the intermediate sheets, or their horizontal extension. Glycine capping agent, that stabilizes nanoparticles and deters their merges, slows down the formation of the aforesaid intermediate. Lowering the initial supersaturation of MgCO_3_, on the other hand, alters the size of the nanoparticles, but does not affect the formation kinetics of “Sheet-like intermediate → Final crystals” transformation. Simultaneous usage of capping agent and spray dryer seems to be an ideal method for the production of nano MgCO_3_ from the aforesaid reaction.

## Introduction

Nesquehonite is a naturally occurring hydrated magnesium carbonate (MgCO_3_·3H_2_O) mineral, that is also produced synthetically due to its large consumption rate^1–4^. The mineral was named after its discovery in a coal mine in Nesquehoning, Pennsylvania in 1890 ^5,6^. Nesquehonite has found applications in paints, paper, coatings, pharmaceuticals, fire retardants, and nuclear waste disposal^7–9^, as well as CO_2_ capturing^10–12^.

Industrial route for the production of Nesquehonite involves reaction of magnesium-containing brines (e.g., magnesium sulfate and magnesium chloride) with a soluble carbonate (e.g., Na_2_CO_3_ or (NH_4_)_2_CO_3_)^13^.

**Fig. 1:**
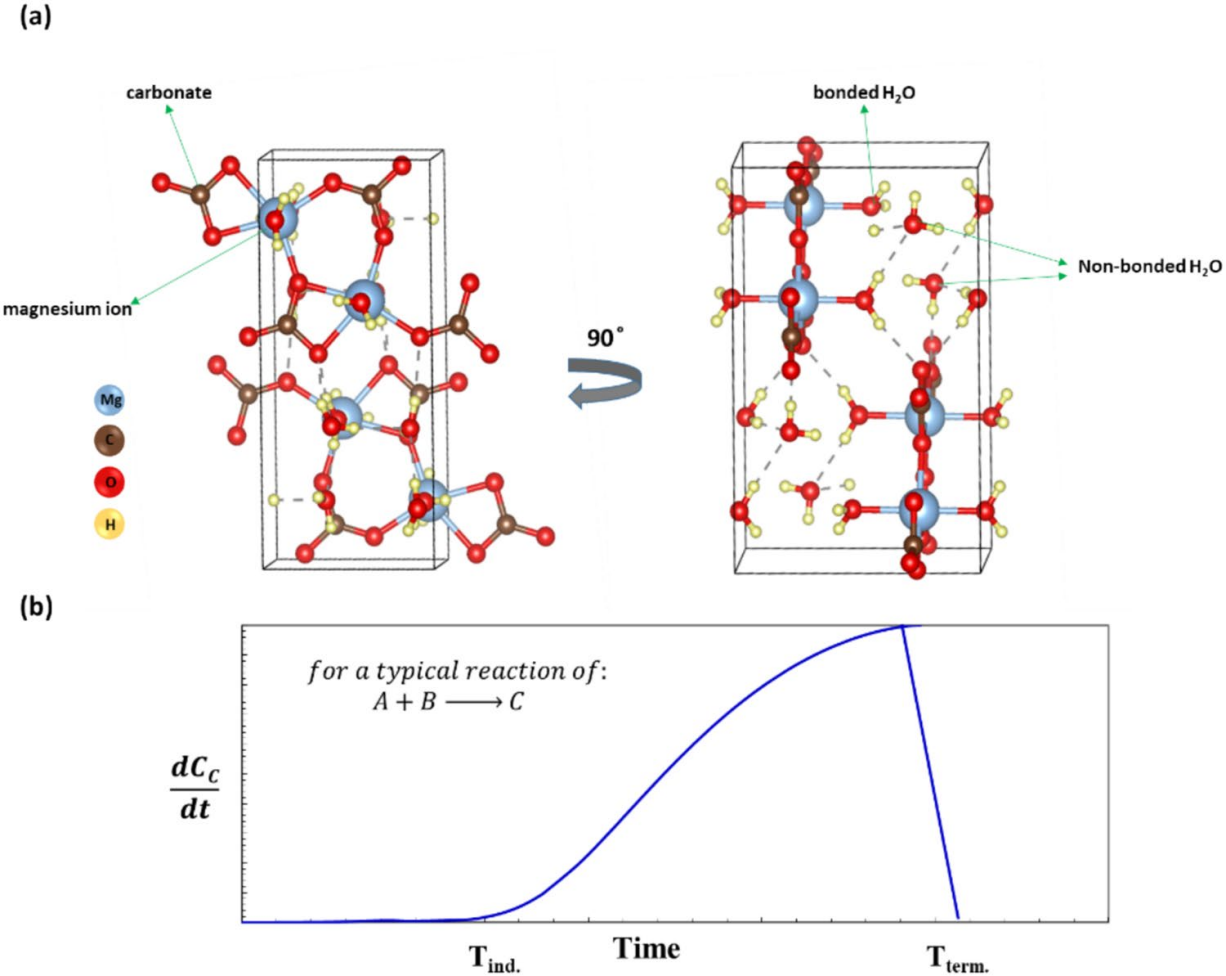
(**a**) Structure of Nesquehonite single crystal: each magnesium atom in the unit cell is bonded to six adjacent oxygen atoms of carbonates and water molecules in the crystal. (**b**), A schematic view of the reaction rate in a typical reaction that exhibit induction period.

Nesquehonite forms perfect crystals with whisker-like morphology^14,15^. Its unit cell (Fig. 1a) consists of a magnesium ion bonded to six oxygen atoms: four from carbonate in equatorial positions and two from water molecules in axial positions^2,16^. These MgO₆ structures creates an extended crystal lattice and polymorphism of the Nesquehonite crystal^2,7^. Alongside with polymorphism, the mechanism of crystal growth and the resulted morphology can determine the macroscopic properties of the final Nesquehonite^17–20^. However, to the best of our knowledge, there are few reports on the mechanism of formation of Nesquehonite crystals from magnesium-bearing brines.

As was mentioned before, one of the most employed routes for the synthesis of Nesquehonite is precipitation of a soluble magnesium salt (e.g., MgSO_4_) with the sodium carbonate. The current authors noticed that after adding the reagents (i.e., MgSO_4_ and Na_2_CO_3_) and elapsing an induction period, an abrupt increase in the surge in viscosity is so significant that it may lead to the partial failure of the agitator system, and should viscosity of the slurry occur (see the attached video in the supporting information). Therefore, this is better to be considered in the reactor design. Simultaneously, other bulk properties of the reaction mixture including pH and temperature undergo the sudden increase after the said induction period. Existence of “induction period” in some chemical reactions is a well-known phenomenon. For those reactions, kinetics is initially slow, but after a certain time, reaction accelerates and in a very short period, it consumes nearly all reactants; afterward, the rate suddenly tapers off (Fig. 1b)^20–24^.

One of the reactions with induction period are catalytic reactions, in which, the added catalyst needs some times to transform to its active form^25–27^. Autocatalytic and highly exothermic reactions may also exhibit induction period^28–30^. Crystallization and precipitation processes sometimes show the induction period, but in quite different manners^20,23^. In this case, either initial nucleation takes long time (induction period) and growth is fast, or transformation of nano nuclei to final crystals faces a kinetic barrier, and needs some time to overcome it.

The current study was undertaken to elucidate mechanism of formation of Nesquehonite in the precipitation process. There are three reasons that makes such investigation useful or necessary for the scientific audience:

1. Phenomenon of induction period is encountered in many precipitation systems, and clarifying its details in an industrially important process (i.e., Nesquehonite synthesis) sheds light to better understanding of it. Note that like all physicochemical phenomena, a complete understanding of induction period, needs mathematical simulation in atomistic and mesoscale levels. But, before reaching that point, a qualitative yet accurate description of the microscale formation mechanisms is required.
2. Engineers who design the precipitation reactors, must be aware of existence of induction period, and its associated phenomena particularly the surge of viscosity. This information will help them in avoiding its adverse consequences and reaching a more delicate design.
3. The last, but not least reason is about production of nanosized magnesium carbonate. This material has recently found a great deals of applications in formulations of cosmetics, toothpaste, dusting powder and fireproofing agents. As evident from the results and discussion, methods such as nanoparticle capping can inhibit the growth of nesquehonite nanoparticles after formation, enabling the production of well-shaped nanomaterials with a uniform size distribution.

To arrive on a detailed mechanism of formation, the chronological evolution of crystal morphology was systematically investigated using electron microscopy, X-ray diffraction, and complementary techniques throughout the reaction process. Concurrently, the bulk properties of the slurry were monitored, and through correlation with microstructural changes, a birth-growth mechanism was identified for nesquehonite formation—from initial spherical nanoparticles to the final microscopic rod-shaped layered structure. Meanwhile, the profound effect of the crystal alteration on the bulk properties of the reaction mixture was also addressed.

## Experimental section

### Materials

Sodium carbonate, magnesium sulfate heptahydrate, ethylenediaminetetraacetic acid (EDTA), Eriochrome Black T, acetone and ethanol were the consumed reagents, which were purchased from the Merck AG (Darmstadt, Germany).

### Methods

#### Nesquehonite synthesis

The synthesis of Nesquehonite in the current study was performed through reaction between magnesium sulfate and sodium carbonate solutions:

$${\text{MgS}}{{\text{O}}_{4({\text{aq}})}} + {\text{ N}}{{\text{a}}_2}{\text{C}}{{\text{O}}_{3({\text{aq}})}} + 3{{\text{H}}_2}{\text{O }} \to \;{\text{MgC}}{{\text{O}}_3}\cdot3{{\text{H}}_2}{{\text{O}}_{(S)}} + {\text{ N}}{{\text{a}}_2}{\text{S}}{{\text{O}}_{4({\text{aq}})}}$$

Although this reaction could be carried out in wide ranges of reactants concentrations, information from some industrial users suggested that the optimum combination of reactants was obtained when concentration of subsequent sodium sulfate was 15% w/v. In order to arrive at such concentration, to achieve this concentration, a 30% w/v MgSO_4_ solution was prepared by dissolving 2.49 mol (614.32 g) of MgSO_4_·7H_2_O in 1000 ml distilled water, which was then rapidly mixed with 1000 ml of sodium carbonate solution containing 2.49 mol (264.17 g) Na_2_CO_3_. This moment was considered as time zero (t = 0) for the formation of Nesquehonite. During and after addition of the materials, liquid was stirred by a mechanical agitator, at 700 rpm, and pH, temperature, and Mg^2+^ concentration of the mixture was examined for about 60 min. The results were taken from three replicas.

In order to study the effect of glycine capping agent on the formation and growth of magnesium carbonate precipitates, the same procedure was undertaken, except that 0.249 mol (18.71 g) Glycine, one tenth of the molar amounts of magnesium, was added to the magnesium sulfate solution.

In the third trial, which investigated the simultaneous effects of capping agent and dilution, the aforementioned procedure was repeated, but this time by diluting concentrations of all reagents by a factor of 10.

#### Measurements of magnesium ion concentration

Aliquots of 5 mL were taken from the agitating reaction mixture at different time intervals and filtered quickly. The filtrate was then acidified using concentrated HCl to dissolve any precipitates formed after filtration. The resulting solution was titrated against an EDTA solution in an ammonia buffer (pH = 10.5) to determine the Mg^2^⁺ concentration. Eriochrome Black T was used as the indicator for the analysis.

#### Morphology of the Nesquehonite crystals

Samples from the reaction mixture were taken at different time intervals. To prevent further reaction from taking place, the samples were transferred to a paper filter equipped with a Büchner funnel and quickly washed three times with distilled water, ethanol, and acetone, respectively. They were then quickly dried at room temperature using an air flow to remove reactants and water, thereby terminating any ongoing reactions and crystal ripening processes. Afterwards, the samples were completely dried at room temperature for 24 h. Their microstructural images were obtained using scanning electron microscopy (FESEM, TESCAN.IRA3LMU, Czech Republic).

#### Rheological tests

During the formation of nesquehonite, a noticeable change in the viscosity of the reaction mixture was observed. However, due to the non-Newtonian nature of the fluid under constant agitation, rheological measurements were performed only on the mixture after 60 minutes of reaction, when the reaction was supposed to be completed. To assess the rheological behavior of the slurry, the sample was allowed to rest undisturbed for one hour. Subsequently, 5 g of the mixture was transferred to a rheometer for analysis. Rheological measurements were conducted using an Anton Paar Physica MCR 301 rotational rheometer (Austria) equipped with a parallel plate geometry (25mm diameter, 1mm gap). The sample volume was slightly more than required to fully occupy the gap between the plates, ensuring proper contact. Then, three rheological tests were performed to evaluate the behavior of the slurry: (i) viscosity as a function of shear rate (Fig. 3a), (ii) viscosity over time under a constant shear rate (Fig. 3b), and (iii) a thixotropic recovery test (Fig. 3c). The thixotropic recovery test involved three sequential shear rate steps: an initial low shear rate of 1 s⁻^1^ to simulate resting conditions, followed by a high shear rate of 100 s⁻^1^, and finally a return to 1 s⁻^1^ to assess the structural recovery of the material.

#### Crystalline structure

In order to assess crystalline structures of the precipitate samples, taken at different times of the reaction, the analysis utilized X-ray diffractometry (XRD, Malvern PANalytical X’pertPro MPD, Netherlands) with copper Kα radiation, with the scanning speed of 0.02 degree/min. The crystallinity percentage was calculated using the following formula:

$$Crystallinity\% = \frac{Crystallinity\,Area}{{Crystallinity\,Area + AmorphousArea}} \times 100$$

#### IR and Raman spectra

Fourier transform infrared, and Raman spectra of the Nesquehonite samples were obtained using (FTIR, Perkin Elmer spectrum RX I, USA) spectrometer, and Raman Microscope (Takram N1-541, Iran), respectively. Application of these techniques was essential in identifying variations in functional groups, and chemical structure of the Nesquehonite, during the course of its formation.

#### Thermogravimetric analyses

The last analyses that could provide a better insight about the nature of the formed precipitate were thermogravimetric analysis (TGA), which was obtained using a METTLER TOLEDO TGA/DSC 1 (Switzerland) instrument.

## Result and discussion

Nesquehonite synthesis was carried out through reaction between near saturated solutions of magnesium sulfate and sodium carbonate, in a stoichiometric ratio, at 25 °C. Materials were mixed rapidly to allow for a high level of supersaturation. At the beginning of the mixing, a white precipitate was formed, but the fluid behavior was not changed significantly i.e., it was still a free-flowing liquid. This condition was stable up to 20 min when the mixture started thickening and gradually became a paste-like slurry. In that situation, propeller that agitated the liquid effectively, at the beginning, lost its efficacy, and was able to mix only a small cylindrical space around it. The mixture then, gradually lost its thickness and became a less viscous fluid with non-Newtonian properties, which was thick when stood intact for a few hours, and would become thin again if was stirred. In order to elucidate the underlying mechanisms of the aforementioned phenomenon, changes in Mg^2+^ concentration (in free liquid), pH, and temperature were monitored for about 1 h, from the time of mixing of the reactants.

**Fig. 2:**
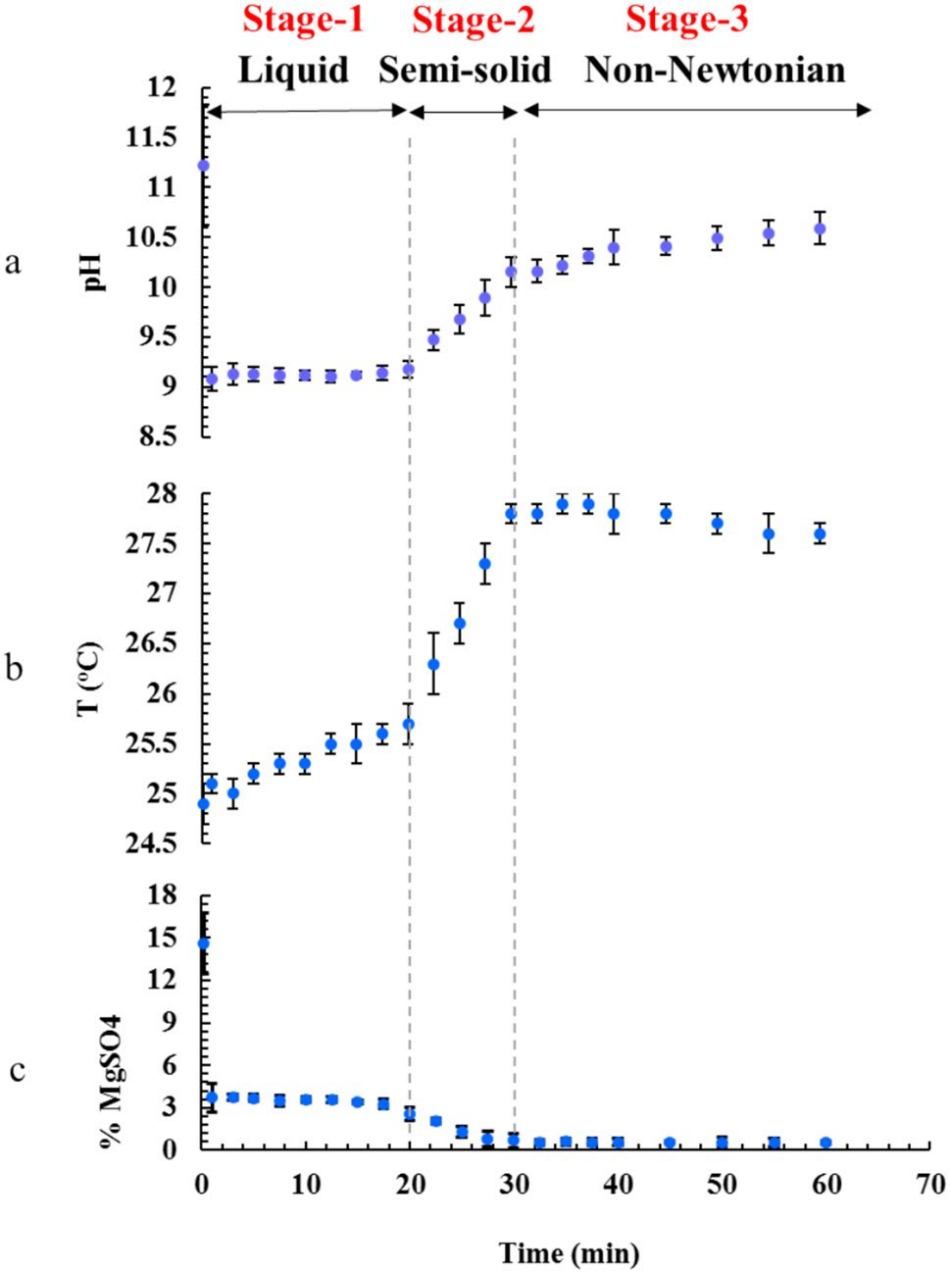
Changes in the (**a**) pH, (b) temperature, and (**c**) magnesium concentration in the solution during the reaction.

Figure 2 displays chronological variations of pH, temperature, and Mg^2+^ concentration, after onset of the reaction. Shortly after the beginning of the reaction, curves of all of the aforementioned parameters leveled off, representing that formation of a relatively stable phase slowed down the process. Interestingly, such conditions did not last long time, and after 20 min, temperature and the pH began increasing, while Mg^2+^ concentration experienced its second round of diminishing. These coincide with the changes in the reaction mixture fluid behavior; i.e., the free-flowing liquid became a paste-like slurry. The simultaneity of liquid thickening with changes of pH, temperature, and Mg^2+^ concentration suggests that the reaction mechanism might include an intermediate step; and the decline in the Mg^2+^ concentration during the phase transformation (20–30 min) demonstrates that a new equilibrium is attained between the newly formed solid phase and the aqueous solution. The simultaneous increase in temperature and reduction of magnesium concentration might be related to this transition to more stable species (with lower free energy and lower water solubility). Also, the simultaneous increase in pH might be the result of a reduction in free water, likely due to gel or crystal formation. After 30 min, the paste-like slurry gradually lost its viscosity and became a non-Newtonian liquid. From 40 min onwards, only small changes were observed in the values of detecting parameters, and the reaction was considered complete at 60 min.

**Fig. 3:**
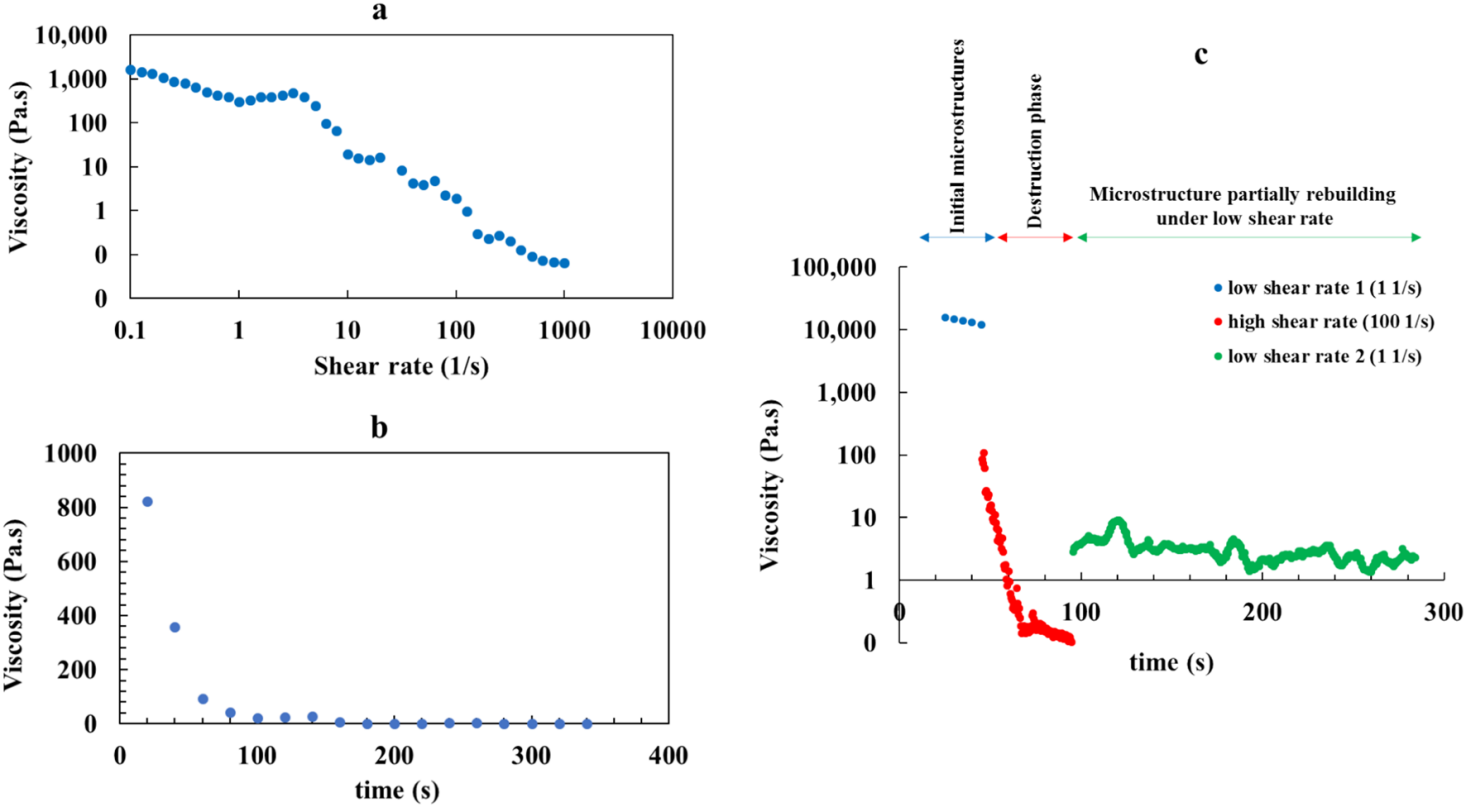
Rheological analysis of the non-Newtonian fluid of the reaction mixture containing final product. (**a**) Flow curve representing changes in the viscosity over different shear rates; (**b**) viscosity over time on the constant shear rate of 10 S^-1^; (**c**) thixotropic recovery test.

In order to identify type of the non-Newtonian liquid product, the final reaction mixture (i.e., after 60 min) was subjected to rotational rheological analysis (Fig. 3). According to Fig. 3a, by increasing the shear rate, viscosity of the sample decreased; i.e., sample indicated a shear thinning property. In another experiment, viscosity of the product solution was examined in the constant shear rate of 10 s^-1^. Results that are presented in Fig. 3b, reveals that under constant shear rate, viscosity of the sample reduced over the time. Combining the results of the two experiments, suggests that the final mixture was a *thixotropic* fluid^31^. In order to determine if the thixotropic behavior were reversible, a set of recovery test was performed. The liquid product was subjected to a three-phases shear loading; initially a low shear rate (1 s^-1^) simulated the resting state of the liquid; in the second interval a high shear rate (100 s^-1^) was used, and eventually, in the third step, a low shear rate (1 s^-1^) was applied again. As Fig. 3c clearly demonstrates, in the second interval a dramatic decrease in the viscosity was observed; which was followed by a significant increase when the low shear rate of third phase was applied.

These decrease and increase in the viscosity curve represent the typical behavior of a reversible thixotropic fluid which corresponds to the breakdown of its microstructure, and its rebuilding, respectively^31,32^. It is noticeable that full recovery of the thixotropic materials may need a long resting time under a stress-free condition. Consequently, the final liquid was left stagnant overnight, and it restored its initial paste-like form, proving its thixotropic nature.

Based on the previous arguments, visual observations and rheological study, the following three-stages mechanism for formation of Nesquehonite was put forward. The first or precipitation stage: occurs immediately after mixing of the reactants. This stage is associated with a high level of supersaturation, that leads to formation of tiny nanoparticles, all across the vessel. In the second stage (between 20 to 30 min) a phase transition takes place that changes the microstructure of precipitate, and in the final stage, the phase transition is completed.

**Fig. 4:**
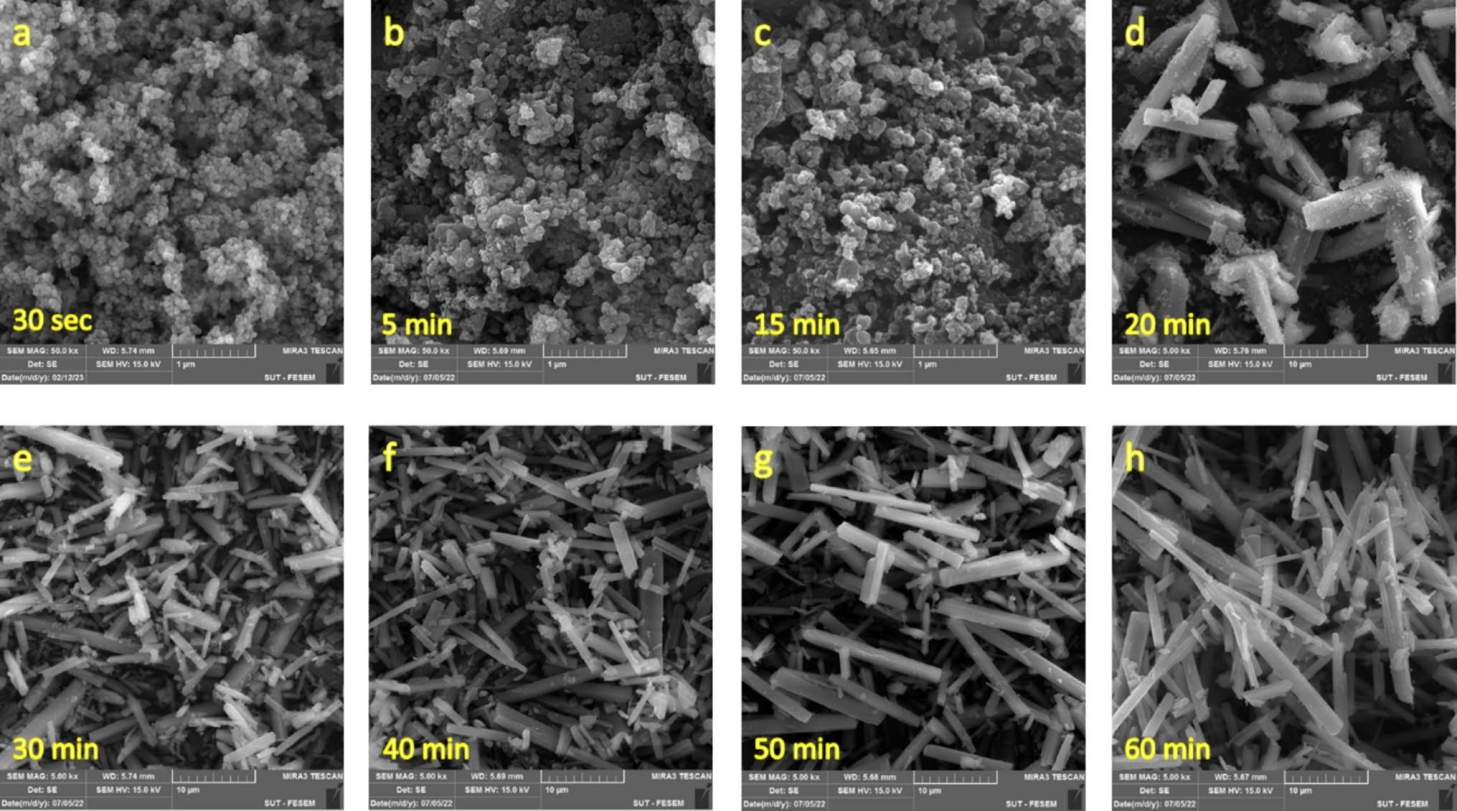
SEM image of samples taken from reaction mixture in different time intervals.

To further elucidate the underlying interactions in the formation of Nesquehonite, variation in microstructure of the precipitate samples was examined using the SEM image (Fig. 4). According to Fig. 4a–c, at initial stages of reaction, semi-spherical and amorphous particles with sizes around 100 nm are formed. It should be noted that the timeframe of these images corresponds to stage (I) of the reaction in Fig. 2, when the reaction mixture is still a free-flowing liquid. In the middle stages (Fig. 4d, e), a phase transitions occurs and most of the particles are transformed to whisker-like crystals with the average length 10 μm, though some keep their initial semi-spherical and amorphous shapes. This stage is superimposable on the stage (II) of the reaction in Fig. 2, when the reaction mixture is semi-solidified afterward. In the final stage (Fig. 4f–h) there is no semi-spherical and amorphous particle visible and only long whisker-like crystals exist; this is equivalent to the timeframe of stage (III) in Fig. 2, when the reaction mixture re-fluidized and forms the final viscous liquid product. Briefly speaking, SEM images show that stage (I) is nucleation stage; in stage (II), the nuclei are converted to the whisker-like crystals, and crystallization is completed in stage (III). Also notable is the fact that viscosity of the reaction mixture is higher in stage (III) than stage (I); possibly because motion of the long rod-shaped crystals (in stage III) with high population in the liquid is spatially more hindered than the tiny spherical particles (stage I). Apparently, viscosity of liquid in stage (II) is higher than stage (III), but this deduction is seeming to be due to thixotropic nature of the whisker-like final product. Indeed, according to the previous discussion (see Fig. 3), slurry in stages (II) and (III) is a thixotropic fluid, whose viscosity decreases over the time. Thus, the lower viscosity of stage (III) is arisen from the longer period, it has been subject to the shear action of propeller^31,33^.

**Fig. 5:**
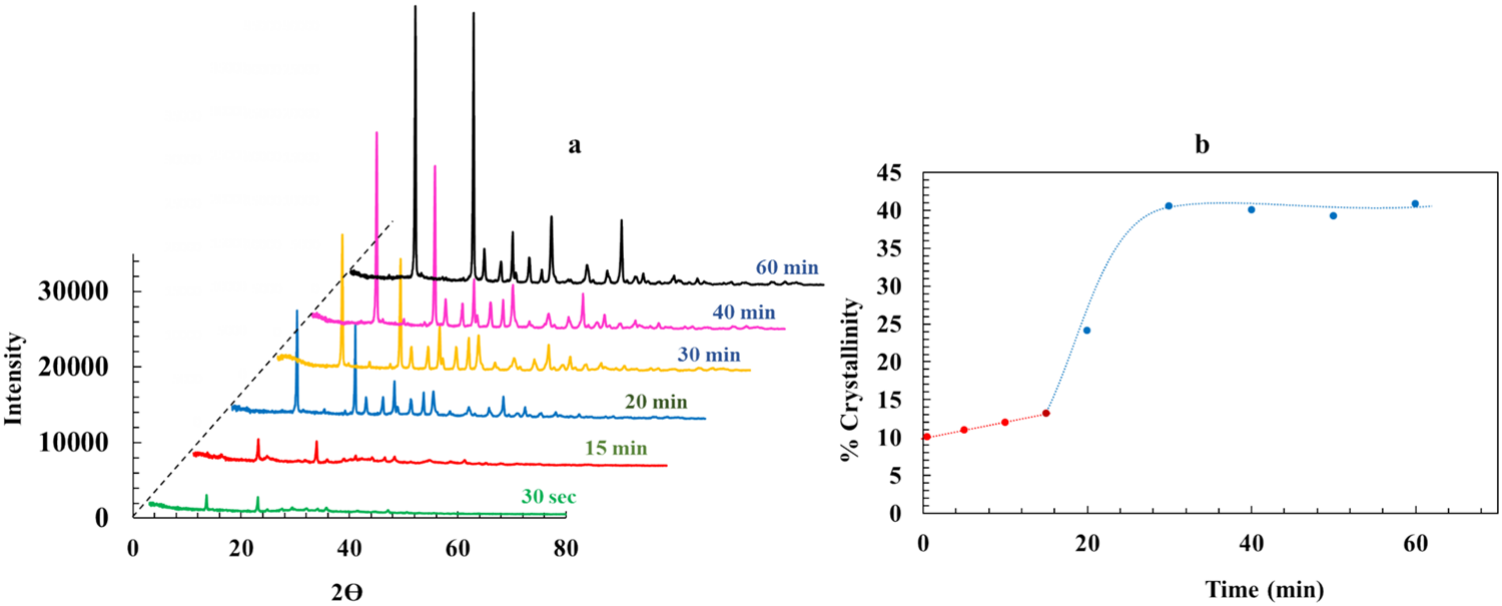
(**a**) X-ray diffractions of different samples taken in different times of reaction. (**b**) Change in the crystallinity percent during the reaction.

Another analysis that provided a deeper insight about the polymorphism of the Nesquehonite crystals during their formation was X ray diffractometry. Figure 5a presents the XRD patterns of precipitate samples collected at various time intervals. The results indicate no detectable change in polymorphic form over time. Instead, a progressive increase in crystallinity was observed, suggesting that crystal growth was the dominant process throughout the reaction^34–36^. Percentage of crystallinity of the Nesquehonite particles was calculated (Fig. 5b). The close agreement between corresponding parts of Figs. (2) and (5b) verifies the existence of a three-stages formation mechanism for Nesquehonite. Part (1) in Fig. (5b) with its nearly constant and low crystallinity presents stage (I), when the principal part of precipitate was in the amorphous (i.e., nano) form. Similarly, ascending part (2) and flat part (3) in Fig. (5b) correspond to stages (II) and (III), when the amorphous particles transform to the more stable crystalline Nesquehonite, and thus give rise to a sharp increase in the crystallinity percentage.

It should be noted that the marked rise of crystallinity in Fig. 5, is concurrent with the other changes including reaction temperature, viscosity, Mg^2+^ concentration and pH. Among them, the simultaneous increase of temperature and crystallinity, is of particular importance, as it reveals the exothermic nature of the phase transformation, leading to the thermodynamically more stable Nesquehonite crystals.

**Fig. 6:**
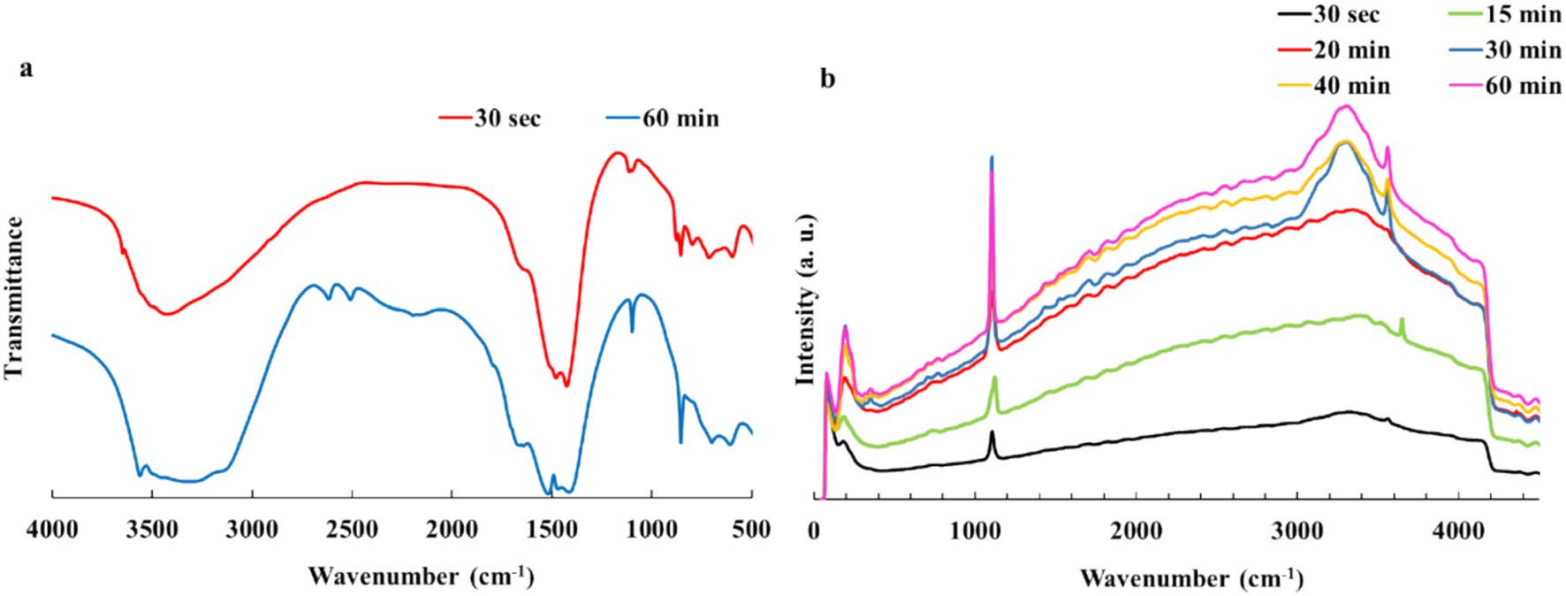
(**a**) IR spectrum of samples at the initial and final stages of the reaction (b) Raman spectrum from different intervals show considerable change in the intensity and the pattern.

To further support the suggested mechanism of the Nesquehonite formation, a combination of IR and Raman spectroscopies were used (Fig. 6). The IR spectra present peaks in about 610 and 700 cm^-1^ which are related to MgO bending, 855 and 1100 for asymmetric and symmetric stretching of CO_3_^2−^. Also, peaks at 1410, 1473, 1518 cm^-1^ are due to asymmetric stretching of CO_3_^2−^ carbon–oxygen bonds; peak in 1651 is arisen from HOH bending and peaks in 3140, 3360 and 3560 are related to O–H stretching of Nesquehonite H_2_O. Therefore, the IR spectra show that from the beginning of the reaction, some magnesium carbonate and Nesquehonite precipitate in the mixture. This is in accordance with the results of the XRD study. In the Raman spectra, peaks that appear at 720, 784, 1110, 1433 and 1553 are representing the asymmetric stretching vibrations of carbonate^5,37^. Meanwhile, the small peak in 1720 cm^-1^ is due to of C = O stretching mode. Bending vibrations of H_2_O and a symmetric stretching of OH group of crystalline water molecule are observed in 3132, 3337 and 3566 cm^-1^ respectively^5,34,37,38^. Figure 6b shows a rise in the intensity of Nesquehonite crystalline water peak intensity in the range of 3000–3600 cm^-1^ as the Nesquehonite crystal formation proceeds. Since at the start of the reaction, magnesium carbonate is mainly precipitated in the amorphous form; the crystalline water’s peak is very weak. Amorphous magnesium carbonate particles transform into crystalline nesquehonite by incorporating water molecules into their crystal structure, which is reflected by an increase in the intensity of the 3000–3600 cm⁻^1^ peak. The observed increase in the pH of the reaction mixture in Stage (II) (Fig. 2b) might be due to this water absorption as will be explained, shortly. As the Nesquehonite crystals are formed, they consume water molecules by using them in their crystal structure.

**Fig. 7:**
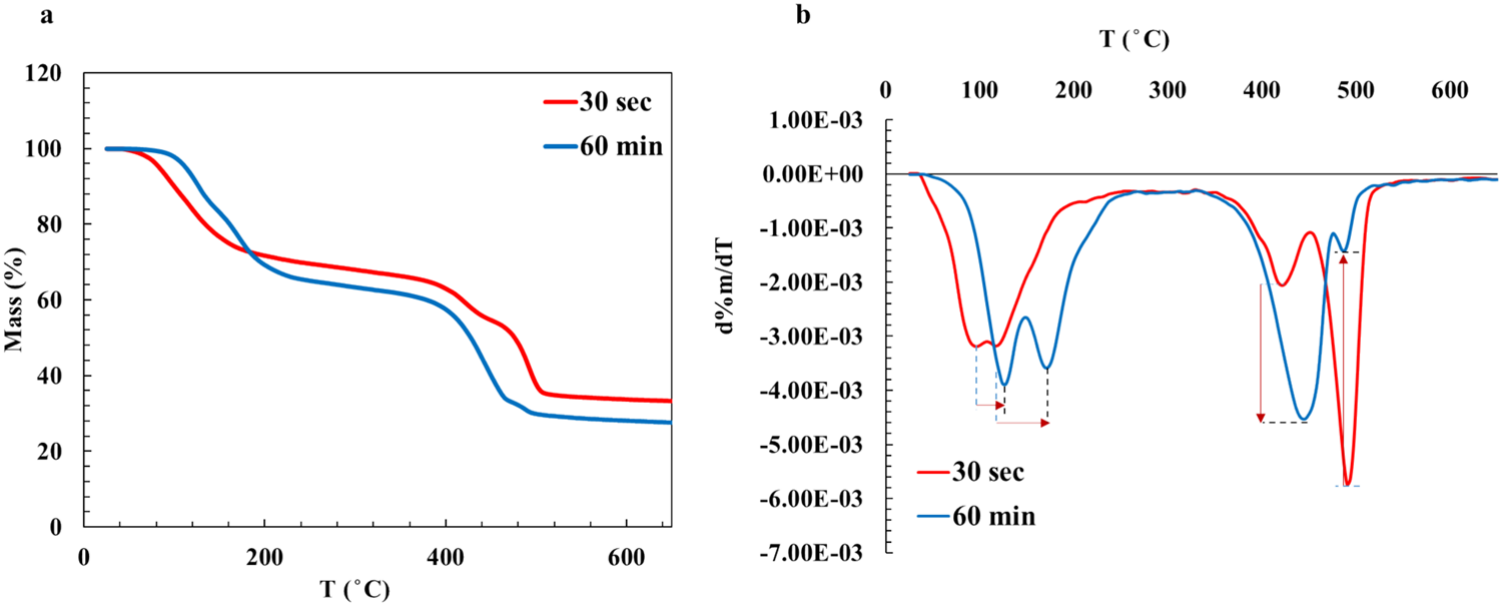
Thermogravimetric analysis of the samples at the beginning and the end of the reaction. (**a**) TGA; (**b**) DTG represents significant difference in the decomposition pattern.

Figure 7 displays the results of thermogravimetric analyses of the precipitate samples, taken at the stages (I) and (III) of the Nesquehonite formation. In the DTG curves (Fig. 7b), the first two peaks of weigh lose (at about 150 °C) are related to the water loss. The third and fourth peaks (both around 450 °C), present different stages of MgCO_3_ decomposition^39–41^. According to Fig. 7b, with elapsing time of the Nesquehonite formation, from 30s to 60 min, dehydration temperature of MgCO_3_·3H_2_O (98 and 113 °C) are shifted toward the higher temperatures (130 and 175 °C) suggesting that the water molecules are engaging more and more in the crystalline structure.

Additionally, two adjacent peaks associated with the decarbonation (i.e., CO_2_ release) of MgCO_3_ show significant changes as nesquehonite formation progresses. In the sample collected at the early stage of the reaction (30 s), the majority of MgCO_3_ decomposed at 490 °C, with a smaller fraction decomposing earlier at 420 °C. In contrast, for the sample taken at the final stage of nesquehonite formation (Stage III), carbonate decomposition primarily occurred at the lower temperature.

**Fig. 8:**
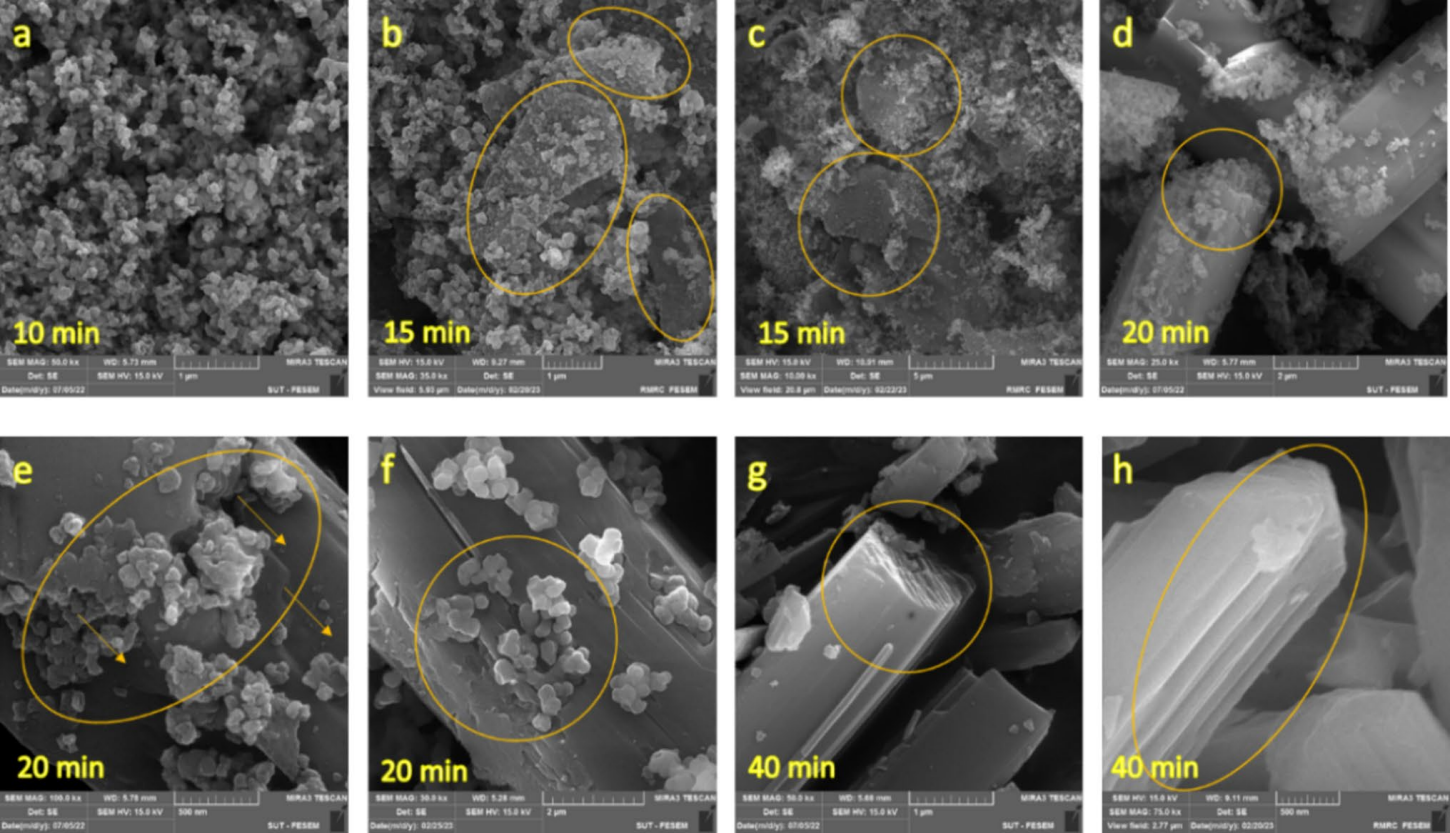
SEM images from different stages of the reaction; (**a**) first stage (10 min); (**b** and **c**) at the start of stage II (15 min); (**d**, **e** and **f**) at the middle of stage II (20 min); (**g** and **h**) final products from stage III (40 min).

In order to more deeply elaborate mechanism of growth of the crystalline Nesquehonite, samples taken before, during, and after phase transformation at Stage (II) were examined using SEM (Fig. 8). At the stage (I), small nuclei in the form of nanoparticles were observed in the mixture (Fig. 8a); then, at the beginning of Stage (II), nanoparticles formed tiny sheets (Figs. 8b, c). This was followed by stacking the sheets, and emerging of the whisker-like crystals (Figs. 8d, e, f). At the final stage (III), almost no nanoparticle has remained and Nesquehonite crystallization was complete (Figs. 8g, h). Also notable is the conspicuous growth front in Figs. 8e, f.

**Fig. 9:**
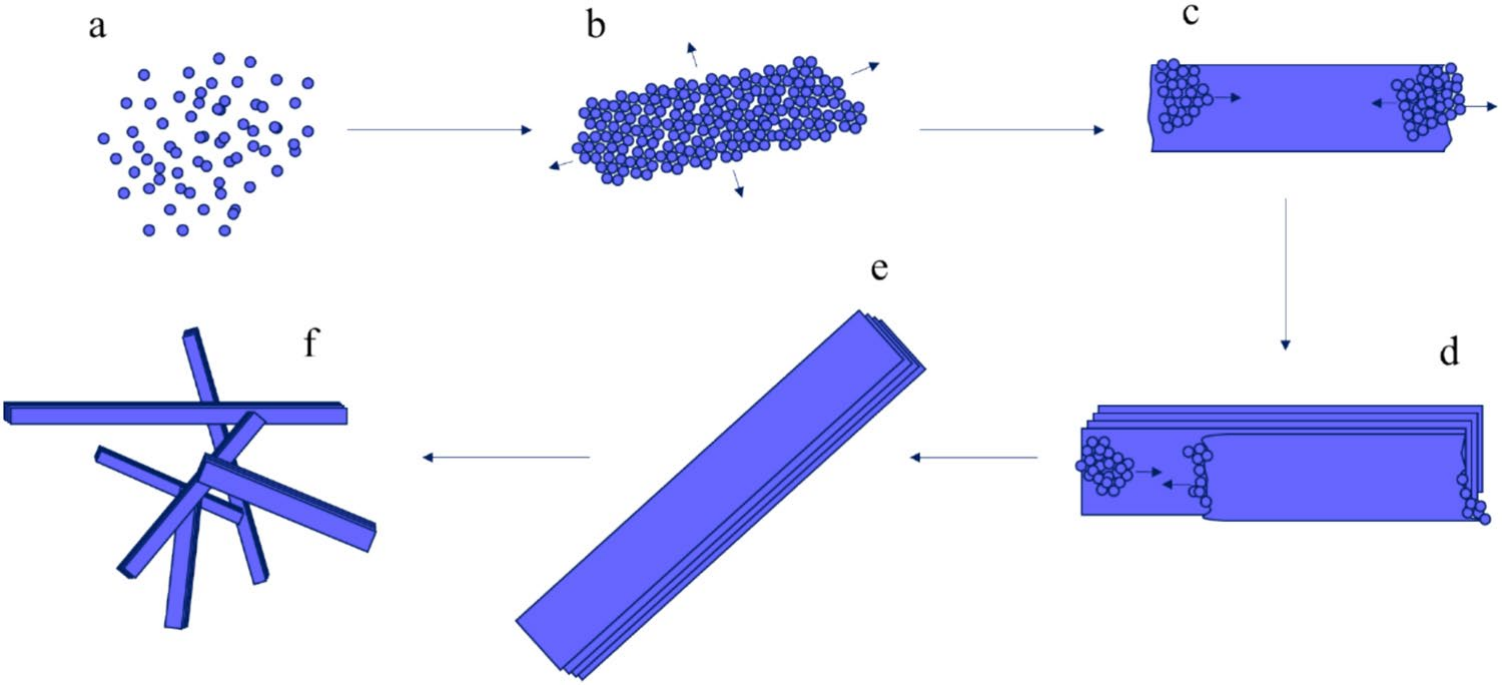
Proposed mechanism for Nesquehonite crystal formation.

Considering the above-mentioned evidence, the following ripening-growth mechanism for the formation of Nesquehonite crystals has been proposed (Fig. 9). At first, the sudden mixing of Na_2_CO_3_ and MgSO_4_ solutions brings about an excessive supersaturation of produced MgCO_3_, which in turn, causes a high rate of nucleation across the liquid volume. Since number of the precipitate nuclei is extremely high, and at the same time, the amounts of the reactants are limited, sizes of the initially formed nuclei is in the nanometer range (Fig. 9a). Elapsing some time, the foregoing nanoparticles merge together to form nanosheets (Fig. 9b), which themselves are the bases of formation of the whisker-like crystals. Evolution of the whisker-like crystals proceeds through both formation of the new layers on the surface, and elongation of the ends of the crystal axis (Fig. 9c, d). The final product is the multilayered whiskers of Nesquehonite crystal (Fig. 9 e, f).

**Fig. 10:**
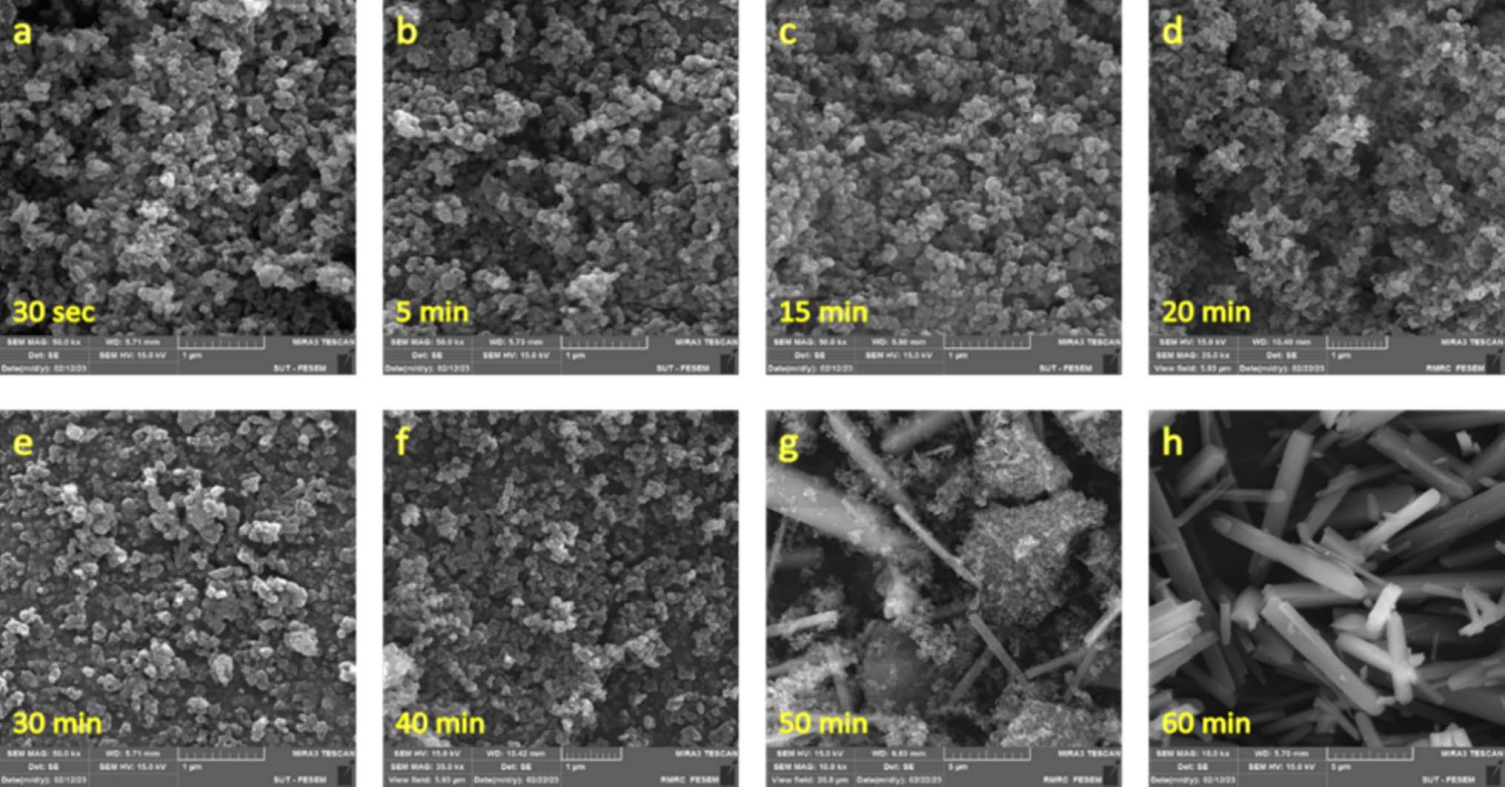
SEM images taken from different time intervals of the reaction in the presence of glycine as a capping agent.

Considering the stepwise changes in crystallinity, temperature, pH and free magnesium ion concentration followed by rapid change to mature whisker like crystals, we suggest that the formation of sheet like intermediate is rate determining step in the growth mechanism. One way to examine the proposed mechanism was to interrupt formation of the intermediate phase in the stage (II) through stabilizing nanoparticles in formed in stage (I). This stabilization achieved using simple capping agent (e.g., glycine). Capping agents are materials that can interact with the surface of the nanoparticles and prevent their agglomeration^42–46^. In this study glycine, which has been used as capping agent to stabilize different nanoparticles, has been used for this purpose^47–50^. If crystal ripening in stage (II) proceeds through formation of the sheet-like intermediate, as a rate determining step, stabilizing of the amorphous nanoparticles should delay production of the fully matured crystals. Therefore, glycine was added to the MgSO_4_ solution, prior to its mixing with the Na_2_CO_3_ solution, with a 1:10 molar ratio of glycine to Mg^2+^. The SEM results revealed that the glycine capping agent did not affect the sizes of the nanoparticles, but it expanded their lifespans by delaying the formation of nanosheets (Fig. 10).

Also notable in Fig. 10 is that after stage (II) was started, the reaction quickly reached stage (III) and glycine was unable to affect the rate of such transformation. In other words, adding glycine only slowed-down “stage (I) → st`age (III)” transformation, and after Nesquehonite sheets were formed, they quickly turned into the fully grown whisker-like crystals. These findings are in close agreement with the proposed mechanism, and emphasize on the importance of the formation of the nanosheets as the critical step of the formation of Nesquehonite crystals.

Supersaturation is one of the most important thermodynamic parameters that has a profound effect on the crystallization process. In order to check the effect of this variable on the growth of the Nesquehonite crystals and to evaluate whether the intervention of sheet formation using glycine can result in similar observations experimental conditions were replicated with the same (glycine/Mg^2^⁺) ratio using tenfold more dilute solutions.

**Fig. 11:**
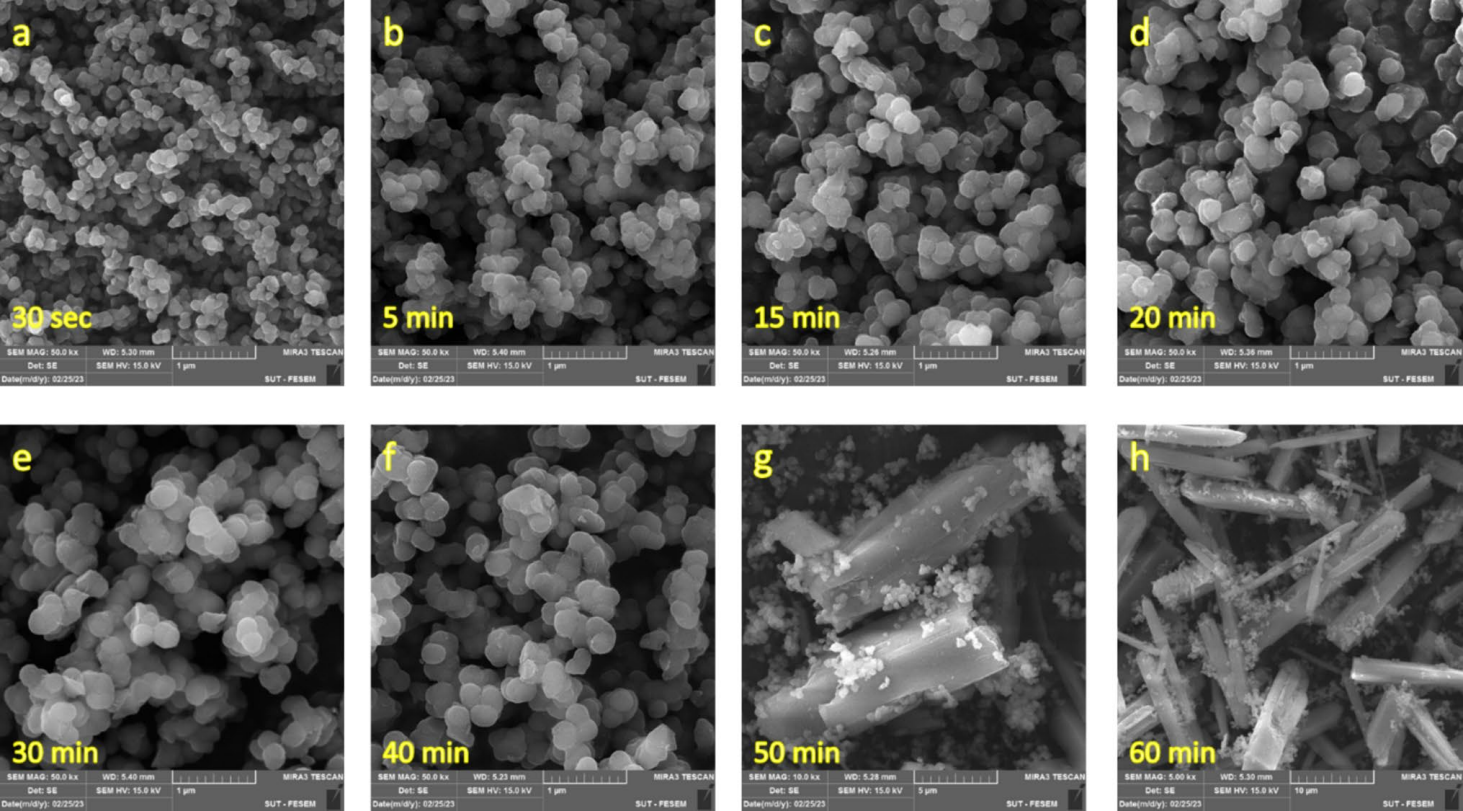
SEM images taken from different time intervals of the reaction in the presence of glycine and at lower supersaturation (10 times dilution of the reaction mixture).

The SEM images of this low supersaturation case are depicted in Fig. 11. The results show that nanoparticles are relatively larger in comparison to those experiments with higher supersaturation. This observation is consistent with the results of the previous studies, in which the lower supersaturation yielded the larger nanocrystals^51–53^.

As observed in Fig. 11, neither the time of appearing the sheet-like intermediate, nor period of completion of the final crystals changed noticeably, in the reduced supersaturation (compare panels g & h of Fig. 10, 11).

It seems that capping agents (e.g., glycine) essentially slow down kinetic of the phase transformation from nanoparticles to the sheets (from 20 to 50 min). But, once the sheet-like intermediate was formed, its conversion to final crystals takes almost the same amounts of time in the presence and absence of capping agents, and in high and low supersaturation (see Figs. 10 and 11). Kinetically speaking, since formation of the nanoparticles is nearly instantaneous, and on the other hand, the sheet-like intermediates react to form the whisker-like crystals, as soon as they are formed, transition from Stage (I) to Stage (II) (i.e., formation of the sheet intermediate) shall be regarded as the rate-determining step of the whole reaction. Noteworthy that stopping the Nesquehonite formation in its nucleation step, or in other word, preventing growth of its nanosized particles can be of high importance, because nano magnesium carbonate has a great deal of applications especially in the health and cosmetic sector^3,4^.

## Conclusion

Nesquehonite or in other word tri-hydrated magnesium carbonate is one of the most significant compounds of magnesium, which alongside with the direct applications, acts as a precursor for production of other magnesium derivatives (e.g., MgO). Synthetic Nesquehonite can be prepared by precipitation of a soluble magnesium salt (e.g., MgSO_4_) with the sodium carbonate solution. Observation revealed that after adding the raw materials, there is an induction period, after which an abrupt surge of viscosity occurs.

In order to disclose the mechanisms of nucleation, growth and ripening of the Nesquehonite crystals, the reaction mixture was scrutinized using a variety of techniques, including SEM, XRD, IR, Raman, and TGA. Such relatively extensive investigation, eventually directed us to the following overall mechanism. At first, under the high supersaturation circumstance, which is created by the sudden mixing of the whole reagents’ volumes, the nano-sized spherical nuclei of magnesium carbonate are formed, nearly instantaneously. Next, those nanoparticles gather slowly, interact with each other, and form the sheet-like intermediate. Completing this stage takes about 20 min, and the induction period that is observed in the laboratory before escalation of the viscosity can be attributed to this slow process. Large crystals of the Nesquehonite are then formed relatively quickly through stacking of the aforementioned sheets, growth of new layers of sheets from particles, or horizontal growth of the layers. The final result is large multilayered whisker-like crystals. In order to find the rate-determining step of the aforesaid mechanism, two additional sets of experiments were designed and performed. In the first series, (MgCO_3_·3H_2_O) nanoparticles after formation, were coated using the glycine. This experiment, clearly demonstrated that stabilizing nanoparticles by using glycine capping agent can slowdown formation of the sheet-like intermediate, and effectively elongate the entire time of the reaction. The second series of experiments addressed the effect of initial supersaturation of magnesium carbonate, immediately after mixing of MgSO_4_ and Na_2_CO_3_ solutions which were 10 times diluted. Observation revealed that reducing supersaturation only alters the size of the nanoparticles before their merge, which results in the sheet-like intermediate, but do not affect formation kinetics of the intermediate, nor it changes required time for “Sheet-like intermediate → Final crystals” transformation. This again proves that the rate determining step of the formation of was the second stage or “formation of the Sheet-like intermediate”.

## Supplementary Information

Supplementary Video 1.

Supplementary Information 1.

## Data Availability

The corresponding author holds the experimental datasets, which can be provided upon reasonable request.
