# Supplementary material for: A chronological study on formation mechanism of nesquehonite from nanoparticles to grown crystals and its application in nanoparticle synthesis
Source: Sci Rep. 2025 Jul 1;15:20956. doi: 10.1038/s41598-025-04662-5 (PMC12216064; doi:10.1038/s41598-025-04662-5)
Supplement: Supplementary file 2 — Supplementary Information 1. [file 41598_2025_4662_MOESM2_ESM.docx]

Supplementary Video Legend:

**Mechanism Behind the Crystallization of Nesquehonite**
